# An Integrated Quantitative Risk Assessment Method for Underground Engineering Fires

**DOI:** 10.3390/ijerph192416934

**Published:** 2022-12-16

**Authors:** Qi Yuan, Hongqinq Zhu, Xiaolei Zhang, Baozhen Zhang, Xingkai Zhang

**Affiliations:** 1 School of Emergency Management and Safety Engineering, China University of Mining and Technology (Beijing), Beijing 100083, China; 2 China Academy of Safety Science and Technology, Beijing 100012, China

**Keywords:** underground engineering fire, fuzzy Bayesian network, energy and barrier theory, preliminary hazard analysis, and bow-tie diagram model, causal relationship, emergency management

## Abstract

Fires are one of the main disasters in underground engineering. In order to comprehensively describe and evaluate the risk of underground engineering fires, this study proposes a UEF risk assessment method based on EPB-FBN. Firstly, based on the EPB model, the static and dynamic information of the fire, such as the cause, occurrence, hazard, product, consequence, and emergency rescue, was analyzed. An EPB model of underground engineering fires was established, and the EPB model was transformed into a BN structure through the conversion rules. Secondly, a fuzzy number was used to describe the state of UEF variable nodes, and a fuzzy conditional probability table was established to describe the uncertain logical relationship between UEF nodes. In order to make full use of the expert knowledge and empirical data, the probability was divided into intervals, and a triangulated fuzzy number was used to represent the linguistic variables judged by experts. The α-weighted valuation method was used for de-fuzzification, and the exact conditional probability table parameters were obtained. Through fuzzy Bayesian inference, the key risk factors can be identified, the sensitivity value of key factors can be calculated, and the maximum risk chain can be found in the case of known evidence. Finally, the method was applied to the deductive analysis of three scenarios. The results show that the model can provide realistic analysis ideas for fire safety evaluation and emergency management of underground engineering. The proposed EPB risk assessment model provides a new perspective for the analysis of UEF accidents and contributes to the ongoing development of UEF research.

## 1. Introduction

Developing underground projects is generally more sustainable, more resilient [1], and more energy-efficient than above-ground projects. Therefore, underground engineering safety is one of the most important tasks for safety managers, but it is still a major problem facing the world [2]. There are some safety challenges in underground engineering, such as the high complexity of infrastructure, closed structures, the high density of personnel, and the difficulty of safe evacuation. The internal safety risk is prominent, whereby fire accidents are the most prevalent underground engineering disasters. Underground engineering fire (UEF) risk assessment, such as high-rise building fire and petrochemical industry fire risk assessment, is the focus and difficulty of modern fire risk rating.

In the underground, enclosed space environment, once a fire occurs, the fire will spread rapidly; if not effectively controlled, the fire will further expand, and personnel escape and rescue relative to the surface space will be much more difficult, resulting in much higher economic losses and casualties than above-ground. According to relevant data and statistics, the death toll of underground space fires is about 5–6 times that of high-rise building fires, and the direct economic loss caused by underground space fires is about 1–3 times that caused by high-rise building fires [3]. One example from 2015 is a fire that broke out in an underground garage of a housing complex in Stuttgart, Germany, setting several cars on fire and resulting in at least 40 people being poisoned by breathing in toxic fumes. Another example from 2003 is a fire that occurred at a subway station in Daegu, South Korea, which killed 198 people and injured 146. In recent years, there seems to be an increasing number of reports about UEFs, and these issues deserve special attention. Therefore, the risk analysis of underground engineering, especially the risk analysis of UEFs, is of great significance in both research and practice.

In order to prevent UEF accidents and reduce safety risks in underground works, there is an urgent need to identify and assess key factors affecting UEF safety risks and to consider the use of more proactive response strategies and more active and/or passive fire systems [4]. Especially for developing countries or regions, it is necessary to pay special attention to UEFs when safety investment in underground engineering safety management is generally insufficient so as to determine the reasonable allocation of safety input and safety actions to be taken [5,6]. For UEFs, some recent studies have proposed different methods to assess UEF security risks [1,7,8,9]. However, most of the previous studies considered the evolution process of a single risk factor, such as the temperature change process and evacuation efficiency. For UEFs involving many dynamic factors, this may not provide a complete picture of the UEF risk. Therefore, the relationship between factors and security risks obtained in previous studies may be biased [10].

This study aimed to analyze the characteristics of UEFs by combining energy and barrier theory, preliminary hazard analysis, a bow-tie diagram, an expert judgment method, fuzzy set theory, the α-weighted valuation method, and a new fire risk analysis, and to propose a probability prediction method for underground engineering based on EPB-FBN. Firstly, the EPB model of UEFs was established from the perspective of energy transfer and security barriers. Directed acyclic graphs (DAGs) of fuzzy Bayesian networks were constructed by transforming EPB to BN. Secondly, a fuzzy conditional probability table was established to describe the uncertain logical relationship between nodes. Combined with expert knowledge and empirical data, the α-weighted valuation method was used to accurately obtain the parameters of the conditional probability table. Finally, BN sensitivity analysis, influence flow analysis, and the deductive reasoning method were used to evaluate the dynamic risk of underground engineering fires, and the key factors and the maximum risk chain were identified. The application results show the feasibility and potential of this method.

## 2. Literature Review

### 2.1. Literature Review of Underground Engineering Fire Analysis Methods

In the past few decades, fire-related topics have aroused widespread interest among researchers. A widely studied subject is the use of risk analysis theories. Various fire risk assessment methods have been proposed, including accident tree analysis, event tree analysis, the Monte Carlo method, regression trees, and the analytic hierarchy process.

Wu and Liu studied the risk assessment of underground coal fires in inland mines within the studied region, established a regional underground coal fire risk assessment index system, and calculated the data layer with a spatial resolution of 1 km × 1 km for each index through the superposition of monitoring data [11]. However, the index system proposed by the authors is based on the assumption that the risk index of each factor contributes equally, which may not be the case in reality. Sipila et al. analyzed the fire risk of enclosed underground storage facilities, constructed an event tree of spontaneous combustion in underground storage based on previous event data, and proposed cost-effective prevention, correction, and other mitigation suggestions to minimize fire risk and improve storage availability [12]. In the QRA modeling of tunnel fires, many input parameters have accidental or cognitive uncertainties. Meng and Qu used the Monte Carlo technique and the extended principle of fuzzy set theory to solve the dependence relationship between variables, proposed the individual risks based on percentiles and the social risk index based on the α-cut, and carried out an actual risk assessment of the KPE tunnel in Singapore [13]. The authors stated that using fuzzy numbers is an effective method to solve uncertainties. He et al. proposed a method for the quantitative assessment of cable fire risk in underground integrated pipeline corridors. This method combines event trees and fuzzy Petri nets to calculate the probability and consequences of cable fire events in integrated facilities, but the authors did not consider the scenario inference of different states of multiple nodes [14]. Kong et al. analyzed the potential fire scenarios caused by the failure of a fire control system by constructing an event tree and obtained the possibility of different fire scenarios and the number of casualties [15]. Zhang et al. established a probability model of the evacuation of fire emergency personnel using the Monte Carlo method and integrated this model into a fire analysis model [16]. Each theory or method has its own characteristics and has made important contributions to fire risk analysis and fire management. However, these traditional methods have certain limitations. Their models are static and cannot incorporate real-world uncertainty, and all of them use binary (discrete) variable states (“yes” and “no”) in modeling. In cases with large numbers of variables, it is time-consuming to calculate the probabilistic importance of each variable, and the conditional dependencies between variables cannot be represented; moreover, when model variables change, all relevant parameters are recalculated, and the model must be rebuilt, which cannot be achieved during the operation probability update [17,18].

In addition, as for the method used to analyze UEFs, some scholars adopted experimental methods to study underground fires, and the characteristics of smoke flow concentrations, temperature distributions, and fire source locations were obtained. Smoke flow is one of the products of underground fires. For underground engineering, the mixing of smoke flow and airflow generated by fires can form fire-wind pressure and cause serious damage. Huang et al. conducted a similar model experiment on the main building of a large underground hydropower station to analyze the characteristics of smoke flow in a fire accident and the influence of the location of the fire source on the height of the smoke flow [19]. If a fire breaks out in an underground space, it will produce a lot of smoke, the flames will spread widely, and the temperature will rise rapidly. Luo et al. established a 1:5-scale model of an underground parking lot by using similarity theory and studied the temperature distribution of the fire in the underground 3D parking lot. The results showed that the heat release rate played a dominant role in the influence of the temperature of each measuring point of the fire in the underground parking lot [20]. At the same time, it is suggested that automatic sprinkler systems have a good extinguishing effect on underground fires. As underground engineering spaces are relatively limited, the toxic and harmful gas produced by fires will seriously threaten the safety of personnel and cause a huge social impact. Long et al. conducted a full-scale fire experiment in an underground station, set up four underground station fire scenes by changing the location of the fire source and ventilation conditions, and analyzed several key fire parameters [21]. On the other hand, smoke flows, toxic and hazardous gases, and high temperatures not only threaten the directly exposed area but also invade adjacent areas and may lead to amplified accident consequences. Liu et al. conducted a full-scale fire experiment in an underground powerhouse of a hydropower station and obtained the variation law of the flue gas temperature with time, the maximum temperature distribution in the upper and lower spaces, the stratification of the flue gas, and the propagation velocity of the flue gas front [22]. Similarity theory and full-scale experiments are powerful tools for studying the combustion and propagation process of underground fires and for conducting scenario analyses of such incidents. However, these field trials are inherently risky, time-consuming, and expensive.

With the development of computer technology, simulation technology and numerical calculation methods are favored by many scholars. Aiming at the evacuation of fire personnel from underground shopping malls, Wang et al. proposed a multi-exit fire-source-location selection model, used the software PyroSim to simulate a UEF, and analyzed the evacuation risk distribution of each area under fire conditions [23]. Wang et al. developed several mechanical ventilation methods and fire locations and used computational fluid dynamics (CFD) methods to study the fire and smoke spread characteristics of natural gas cabins in urban underground utility tunnels [24]. As for the fire problems of underground parking lots, Barsim et al. simulated nine different fire scenarios with a CFD program and conducted simulation experiments of the transient heat release rates of two car fires [25]. Li et al. assessed the ventilation performance of a super-large section of an underground engineering project, and a full-scale 3D CFD model was constructed to simulate indicators such as CO, dust, and oxygen supply in a fire scenario [26]. Simulation is a good way to study the fire evolution process. However, some complex underground engineering scenarios involve complicated modeling processes, large amounts of computation, and inefficient fire emergency management.

The above analysis methods provide support for the study of UEFs, but the safety risk of underground engineering is dynamic; in the absence of a large amount of historical data, the application of BN in UEF analysis is not simple, especially in hazard identification and model building. There has also been some research on the hazards of UEFs, but only some aspects of the hazards have been studied rather than complete hazard identification with a clear hazard representation. Alimzhanova et al. studied the effectiveness of sprinkler systems in car parks; reductions in fatalities, injuries, and property damage were considered as potential benefits after installing sprinklers [27]. Deng et al.attempted to analyze the impact of underground coal fire pollution on the atmospheric environment and the effects of toxic and harmful gases such as CO, CO_2_, SO_2_, and NO [28]. Ju et al. determined a fire risk index system for a subway station through expert consultation [29]. Therefore, to address the identification of risk factors, prediction of consequences, and determination of key hazards for UEFs simultaneously, it is crucial to develop a model that can be used in the hazard identification of UEFs in a comprehensive, detailed manner, which is an important aspect of this study.

### 2.2. Literature Review of Bayesian Network Applications in Risk Assessment

The key challenge in the risk analysis of complex systems is to deal with randomness and ignorance-type uncertainties. BNs have therefore become a popular tool for the risk analysis of complex problems [17]. Early application of BNs can be found in the study of [30], where they pointed out that BNs are considered the most promising technology to support dependability argumentation. Kabir and Papadopoulos presented a comprehensive review of the use of BNs in system safety, realizability, and risk analysis and highlighted the potential usefulness of BN-based approaches over other classical approaches and their relative strengths in different practical application scenarios [31]. Tang et al. studied the safety risks of rural roads, established a Bayesian network with eight nodes, and assessed the safety risks of rural roads by using experts’ judgment on the conditional probabilities of different safety risk factors. The results showed that the proposed Bayesian network could be used as a low-cost solution for security assessment with relatively high accuracy [10]. Cheng and Hadjisophocleous used a Bayesian network to calculate the probability of a fire spreading from the fire source to other parts of the building and set up two scenarios: one without a sprinkler system installed and another with a sprinkler system installed. The results showed that sprinkler systems could significantly reduce the spread of fires [32]. Matellini et al. studied the evolution and development of a house fire by using a Bayesian network and proposed dividing the fire development process into three parts: “initial fire”, “personnel response”, and further fire development [33]. Hanea and Ale used a Bayesian belief network to evaluate building fires and analyzed the characteristics of fires in different stages of the building design process [34]. Gran and Helminen used a BN model for the reliability assessment of nuclear plants [35]. Liu et al. proposed a method for developing a BN model for the risk analysis of subsea blowout preventer stacks in the presence of common cause failures [36]. Hänninen studied the role of Bayesian networks in maritime accident prevention and safety modeling, discussed some of the challenges in applying Bayesian networks, and analyzed the possibilities of combining data with expert knowledge and the ways in which models can be easily updated when more accurate evidence is obtained [37]. In addition, many scholars have used BNs to carry out a significant amount of research in biology, construction, hydrology, supply chains, oil and gas, and other fields [38,39,40,41,42,43,44,45]. However, the above research needs to accurately know the probability value of each variable before using a BN deductive reasoning algorithm for analysis. In practical engineering, it is often difficult to determine the exact probability value of the factors affecting UEFs. In addition, due to the lack of statistical data, it is difficult to accurately represent the probability value of each variable.

## 3. Fundamental Theory

### 3.1. EPB Model

**Figure 1:**
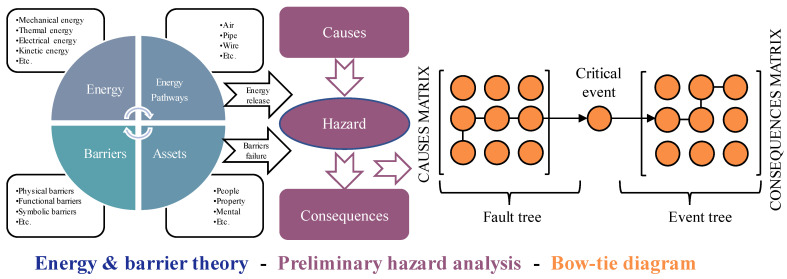
Energy and barrier theory, preliminary hazard analysis, and bow-tie diagram (EPB) model.

There are many factors involved in the UEF process, and they interact with each other. In order to identify these influencing factors timely, accurately, and quickly, we propose the energy and barrier theory (EBT), preliminary hazard analysis (PHA), and bow-tie diagram (BTD) analysis methods. Through the collection of UEF cases and related literature, the occurrence, development, products, and hazards of UEFs were analyzed from the perspectives of energy transfer, barrier failure, and causality. The risk factors of UEFs and their causal relationships were revealed, and the EPB model of UEFs was established to identify the basic nodes (root, intermediate, and child nodes). The EPB model is shown in Figure 1.

**Figure 2:**
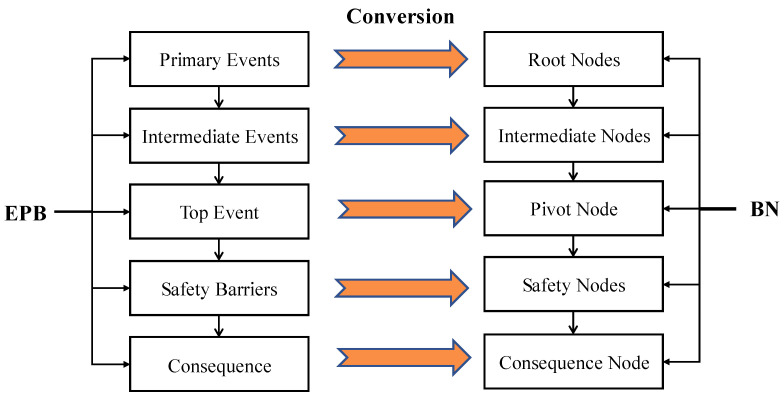
Conversion from EPB to BN (based on Khakzad, 2013 [50]).

(1) Engineering safety science states that there is an accident development process. PHA can be used as a basic method for UEF hazard analysis in this stage. PHA generates a macro-level overview of the hazard categories, occurrence conditions, and possible consequences of a system. The most critical issue is to identify major potential hazards and then analyze their causes and consequences [46].
(2) From the perspective of energy transfer, an underground engineering structure can be regarded as a complex system, and a UEF can be considered a security incident that occurs in this complex system. Therefore, in the study of UEFs, accident causation theory can be applied as the safety principle for analysis. This theory is the basis of hazard identification analysis, and the energy and barrier theory states that accidents occur when dangerous energy is out of control and vulnerable assets have no effective safety barriers [47]. Therefore, a UEF has also released runaway energy. For example, the rotating parts of power equipment in an underground engineering structure rub against each other. This causes sparks, which ignite the surrounding combustibles and start a fire. A fire is a typical accidental release of thermal energy, and uncontrolled thermal energy may injure people and damage equipment. Safety barriers, which include automatic alarm devices, automatic water spray devices, fire doors, fire-extinguishing materials, and explosion-proof layers of power equipment in underground engineering structures, are physical or nonphysical means of preventing, controlling, or mitigating undesired events or accidents [48,49].
(3) BTDs have been well-proven to be reliable and efficient models. BTDs provide a clear view of the causes and consequences of accidents. A BTD is composed of two parts: a fault tree and an event tree; it suffers from the limitations of both constituents. The fault tree is a pre-control process to identify the hazards that may lead to accidents (top event), while the event tree is a post-control process to show the possible consequences of unsafe events.
(4) Finally, we propose a conversion method that transforms the EPB model into a BN model. The conversion from EPB to BN is based on the work of [50]. The BN structure is mainly established by determining node variables and directed edges representing the probabilistic causality between node variables. Through the comprehensive analysis of the EPB model, the occurrence, development, safety measures, products, and hazards of UEFs are identified, which can provide effective prior knowledge for the establishment of directed acyclic graphs (DAGs) of the BN model. The main event, intermediate event, and top event of the fault tree correspond to the root node, intermediate node, and leaf node in the BN, respectively, and the transformation from the event tree to the BN is similar to that of the fault tree. When the developed accident scenario model is complex, some nodes need to be modified for the convenience of calculation and inference. Figure 2 illustrates the steps of converting an EBP model to a BN.

### 3.2. Fuzzy Set Theory

There are many types of uncertainties in engineering practice, which make it difficult to express clear and concise information.

L.A. Zadeh introduced the fuzzy set theory to cope with uncertainty and imprecision [51], which are inherent to human judgment in decision-making processes with linguistic terms and degrees of membership. A fuzzy set is a class of objects with grades of membership. These grades present the degree of stability to which a certain element belongs to a fuzzy set [52]. $\tilde{U}$ is usually used to denote a fuzzy set, where x is characterized by a membership function $F_{\tilde{U}}\left(x\right)$ with an interval of [0, 1]. In fuzzy set theory, there are three ways to transform uncertain numbers into fuzzy numbers: triangular, trapezoidal, or Gaussian fuzzy numbers. In contrast to trapezoidal and Gaussian fuzzy numbers, triangular fuzzy numbers are suitable for the comparison and calculation of many qualitative criteria and are often applied to simple subjective evaluation problems of uncertainty [53]. At the same time, they have the advantages of being easy to understand and having a simple solution process. Moreover, the membership functions of trapezoidal and Gaussian fuzzy numbers are more complicated, and many critical points of membership functions need to be defined.

**Figure 3:**
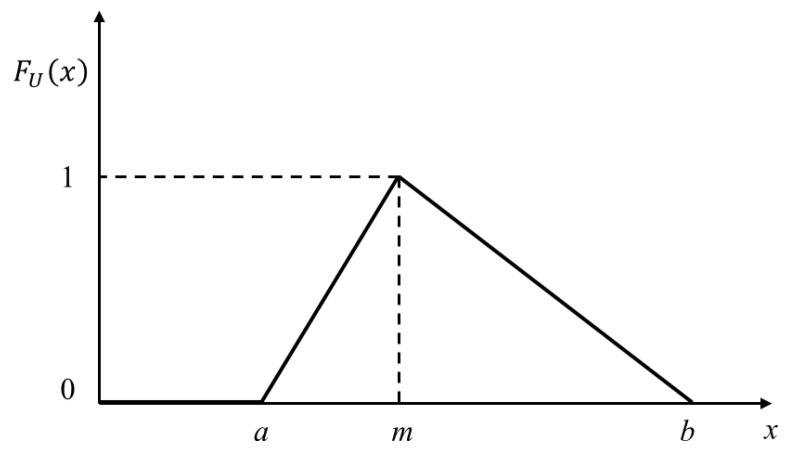
Membership function of a triangular fuzzy number $\tilde{U}$.

The algebra operation of the membership functions of trigonometric fuzzy numbers is simple and widely used. In this study, a triangular fuzzy number is chosen to represent the fuzzy probability of the BN nodes. Figure 3 shows a representation of a membership function of a triangular fuzzy number, and the membership function is given by Equation (3).

.

(1) $$F_{\tilde{U}}\left(x\right)=\{\begin{array}{ll} 0, & x\leq a\\ \frac{x-a}{m-a}, & a<x\leq m\\ \frac{b-x}{b-m}, & m<x\leq b\\ 0, & x>b \end{array}$$

where a and b are the lower and upper least likely values, respectively, represent the mean value.

Assuming there are two triangular fuzzy numbers, namely, ${\tilde{U}}_{1}=\left(a_{1},m_{1},b_{1}\right)$ and ${\tilde{U}}_{2}=\left(a_{2},m_{2},b_{2}\right)$, the algorithm between ${\tilde{U}}_{1}$ and ${\tilde{U}}_{2}$ can be defined by Equation (4):

(2) $${\tilde{U}}_{1}+{\tilde{U}}_{2}=\left(a_{1}+a_{2},m_{1}+m_{2},b_{1}+b_{2}\right)\;{\tilde{U}}_{1}-{\tilde{U}}_{2}=\left(a_{1}-a_{2},m_{1}-m_{2},b_{1}-b_{2}\right)\;{\tilde{U}}_{1}\times {\tilde{U}}_{2}=\left(a_{1}\times a_{2},m_{1}\times m_{2},b_{1}\times b_{2}\right)\;{\tilde{U}}_{1}÷{\tilde{U}}_{2}=\left(a_{1}÷b_{2},m_{1}÷m_{2},b_{1}÷a_{2}\right)$$

At present, fuzzy set theory has developed into a complete theoretical system involving computer science, artificial intelligence, and information processing [54,55].

### 3.3. Bayesian Network (BN)

A BN is a mathematical model that combines graph theory and probability theory [39]. It uses the mathematical language of graph theory to elucidate the problem structure qualitatively. Its topology is a directed acyclic graph (DAG) consisting of a set of nodes (random variables), edges (causal relationships), and a conditional probability table (CPT) [56]. BNs are advantageous for dealing with uncertainty and are flexible inference engines that are widely used in modeling, risk analysis, and reliability and fault maintenance in various scenarios [57].

In this study, the basic definition of a BN is as follows.

Network nodes: The research object of a BN is called the network node, which can be any random variable in the research field. In this paper, each factor derived from EPB model analysis belongs to a network node.

Directed edges: The network nodes and directed edges constitute the topology of a BN. Directed edges between nodes represent causal relationships.

Conditional probability table (CPT): A CPT is mainly used in the calculation of BN reasoning, which reflects the probabilistic relationship between a child node and a parent node, including the probability that the node takes each state.

The joint distribution of BN variables can be decomposed into the product of conditional probability distributions [58]. According to the conditional independence assumption of BNs, let $Parent\left(X_{i}\right)$ represent the parent node of variable $X_{i}$. The joint probability distribution of a set of random variables $U=\left\{X_{1},X_{2},X_{3},\ldots ,X_{n-1},X_{n}\right\}$ can be given using the following chain equation:

(3) $$p\left(U\right)\;=\;P\left(X_{1},X_{2},X_{3},\cdots ,X_{n-1},X_{n}\right)\;=\;\overset{n}{\underset{i\;=\;1}{\prod }}P\left(X_{i}|\mathrm{Parent}\left(X_{i}\right)\right).$$

The main application of BNs is probabilistic updating. When new evidence emerges, other methods, such as fault trees, must build a new model for each piece of evidence, whereas a BN can automatically update the probabilities with limited information. Let *E* denote the newly added evidence in a BN. The updated posterior probability can be given by the following equation:

(4) $$p\left(U|E\right)\;=\;\frac{p\left(E|U\right)\cdot p\left(U\right)}{p\left(E\right)}\;=\;\frac{p\left(E|U\right)\cdot p\left(U\right)}{\sum_{i\;=\;1}^{k}p\left(E|X_{i}\right)\cdot p\left(X_{i}\right)}.$$

p(X∣E) represents the posterior probability of the newly added evidence *E*; p(E∣X)/p(E) is the likelihood function; and p(X) represents the prior probability of node X.

In the traditional BN model, the node probability is usually set accurately. However, in the field of underground engineering, a lack of data or changes in the system environment are often faced, and it is difficult to obtain accurate probability values. Hanss pointed out that fuzzy set theory provides a powerful tool for solving engineering problems under uncertainty and can deal with uncertainty according to fuzzy numbers [59]. Therefore, in addition to proposing fuzzy Bayesian networks (FBNs), some scholars have proposed the application of fuzzy set theory to BN models [60,61,62]. The existing research shows that, under the same model and the same input data, a fuzzy Bayesian network can obtain consistent results, with the method being more universal. Therefore, it can be said that this combination is the inheritance and development of traditional BNs.

## 4. Comprehensive Risk Assessment Model Method Based on EPB-FBN

**Figure 4:**
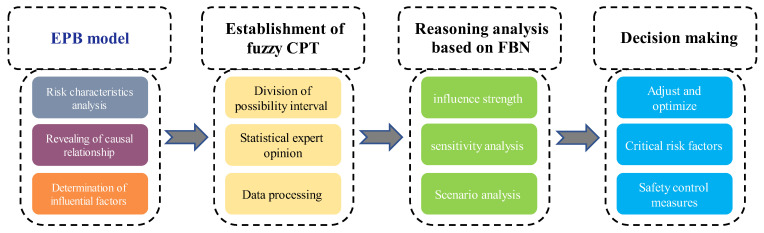
The evaluation process for UEF based on EPB-FBN.

The identification and analysis of the influencing factors of UEFs are the basis of the determination of Bayesian network topology. This method includes four steps: UEF risk factor analysis, FBN establishment, BN node fuzzy probability estimation, FBP-based reasoning analysis, and decision making. The step-by-step evaluation process is shown in Figure 4.

### 4.1. Establish the FBN Model

#### 4.1.1. Determination of Influential Factors and EPB Model

**Figure 5:**
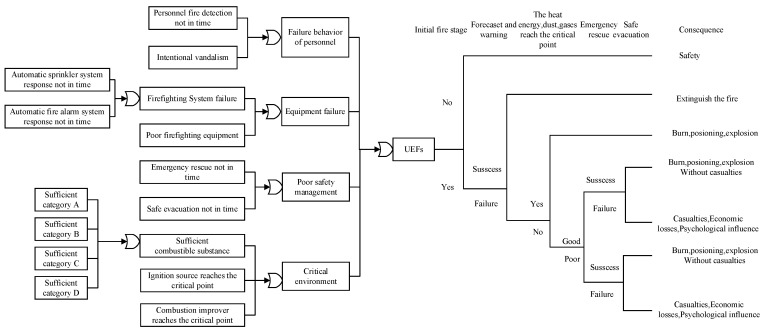
EPB for UEFs.

In engineering applications, the UEF process contains many influencing factors, and these influencing factors interact with each other. In order to quickly and accurately identify these influencing factors, an analysis process is proposed according to Section 2.1, and the EPB model of UEFs is established. The EPB model of UEFs is shown in Figure 5.

The characteristics and hazards of underground engineering fires are mainly manifested in the following aspects: (a) The fire develops rapidly. The environment of underground engineering areas is complex, and the energy of smoke and heat exhausts is limited. Once a fire occurs, the combustion products will rapidly spread around and accumulate with the fire smoke. (b) High temperature and concentration of flue gas. Under poor ventilation conditions in underground engineering areas, generally for natural ventilation or forced ventilation, some combustible material in the fire burns in a hypoxia state, and the poisonous and harmful ingredients are contained in the products of combustion. As the smoke accumulates in the space of the personnel, it can cause poisoning, suffocation, burns, fever, hazards, fear, etc. (c) Difficulty in evacuating personnel. In underground engineering, the density of personnel is high, and the environmental structure is complex. After a fire occurs, the direction of smoke diffusion may coincide with the personnel evacuation passage due to the directionality of heat flow, which brings a serious threat to the safe evacuation of personnel during a UEF. (d) Firefighting is difficult. UEFs can be accompanied by power outages and a lot of smoke, making it difficult for rescuers to determine where the fire is and how it is burning. At the same time, in firefighting, the fire smoke diffusion direction and rescue direction are opposite to each other, and a large amount of high-temperature smoke is faced head-on, increasing the difficulty of rescue. The fire-extinguishing system installed in underground engineering areas may face the problem of failure or response delay. In addition, it is difficult for large firefighting equipment from outside to enter the underground engineering area. In terms of comprehensive underground fire characteristics, in the event of a fire, the underground space temperature rises rapidly, the fire can be destructive, and the fire risk will be more serious than that of an above-ground fire, disrupting the normal order of production and living. Tragic fire accidents create fear and can even lead to major accidents where groups of people can be injured or even killed.

#### 4.1.2. Determination of DAG

**Figure 6:**
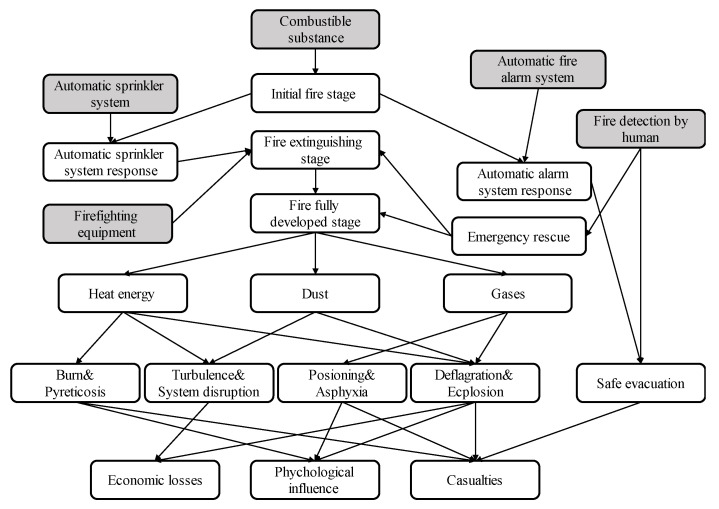
DAG of UEF.

According to the above EPB model analysis and the conversion rule, the BN structure of the established UEF is shown in Figure 6. As can be seen, the DAG of the UEF contains a total of 22 nodes (5 root nodes, 14 intermediate nodes, and 3 leaf nodes). Table 1 presents the status of each node, and the following subsections briefly describe the 22 nodes.

Root nodes

1. Combustible substance. The National Fire Protection Association classifies fires into four categories according to the type of combustible. According to the fire field fire extinguishing agent selection and material combustion characteristics in the field of fire protection, it can be divided into category A fires (wood, rubber, coal, and other solid combustion containing carbon), category B fires (gasoline, kerosene, diesel, methanol, ethanol and other combustible liquid combustion), category C fires (natural gas, methane, acetylene, coal gas, and other combustible gas combustion) and category D fires(sodium, potassium, magnesium and other combustible metals combustion). UEFs are generally category A or category C fires.
2. Automatic sprinkler system. This root node indicates that a fire sprinkler system is installed in the underground engineering area. An initial fire is small and unstable, and an automatic sprinkler system can effectively control and extinguish such a fire.
3. Automatic fire alarm system. This root node means that an automatic fire alarm system is installed in the underground engineering area. Relevant Chinese standards require the installation of fire alarm systems in underground engineering structures. Due to the harsh underground working conditions, there are interferences causing automatic alarm systems to enter a state of delayed response and failure, such as smoke, temperature, and humidity. In the initial fire stage, the normal operation of automatic alarm systems plays a key role in the safe, timely evacuation of personnel.
4. Firefighting equipment. According to relevant Chinese standards, an underground engineering structure must be equipped with firefighting equipment, firefighting pipelines and branches, firefighting material warehouses, and fire-resistant doors.
5. Personnel fire detection. This root node states whether the personnel in the underground engineering area can detect the fire in time. This plays a key role in the safe, timely evacuation of personnel. Moreover, prompt discovery allows personnel to immediately report the fire situation to the emergency rescue department and request professional assistance.

Intermediate nodes

6. Initial fire stage. This node denotes the first 15 min of a fire, where the combustion range is small, the heat energy is not strong, the fire develops slowly, and the smoke has high flow but slow spreading speed. Therefore, the initial fire phase is the best time to extinguish a fire.
7. Automatic sprinkler system response. An automatic sprinkler system can effectively control the development of a fire. If a sprinkler system responds in a timely manner, especially within 30 s of the initial fire phase [6], then the fire is more likely to be extinguished or controlled.
8. Automatic fire alarm system response. An automatic alarm system is an important element in fire detection [6]. If an automatic alarm system responds within 30 s of fire detection, then evacuation can be conducted safely and effectively, thereby reducing casualties.
9. Emergency rescue. Emergency rescue is vital during fires in underground engineering structures. Emergency rescue forces are characterized by occupation, technology, and militarization and can greatly reduce the casualties and property losses caused by disasters in accident rescue. In this paper, emergency rescue forces are assumed to conduct mainly firefighting and personnel evacuation.
10. Fire extinguishing stage. Various measures can be implemented to extinguish a fire in its initial stage. In this paper, this node represents the effectiveness of fire suppression during the initial fire process.
11. Full fire development stage. This stage of fire development is the most dangerous and destructive. Heat energy, toxic and harmful gas components and concentrations, and flue gas turbulence all reach their peaks. In this stage, the combustion will produce sudden changes at any time. If there is explosive gas, instantaneous deflagration and explosion will occur. This node indicates that the fire is not effectively controlled.
12. Heat energy. Due to the narrow flow space of the smoke, the heat energy generated by the combustion cannot be diffused, and the temperature of the smoke flow can reach hundreds or even thousands of degrees.
13. Dust. Combustion produces large amounts of dust and water vapor, which mix with the wind flow over the fire area to form a fire plume.
14. Gases. The gas products generated by fire combustion are mainly CO and CO_2_, and the smoke also contains small quantities of water vapor, methane, acetylene, hydrogen, and heavy hydrocarbons. Due to the different burning intensities, the concentration of toxic and harmful gases in such smoke is different.
15. Burn and pyreticosis. The combustion produces considerable heat, which increases the temperature of the air stream. People who enter the high-temperature plume area are prone to burns. Furthermore, by staying in the plume (whose temperature is higher than their body temperature) for a long time, they become prone to pyreticosis.
16. Turbulence and system disruption. The heat energy generated by the fire is converted into fire wind pressure, which provides additional ventilation power to the original ventilation system and changes the structure of the original ventilation system to make the wind flow disorder.
17. Poisoning and asphyxia. During a fire, combustibles burn to generate a large amount of toxic and harmful gases, such as carbon dioxide, carbon monoxide, and hydrogen. Combustion increases the concentration of toxic and harmful gases in the wind stream and decreases the concentration of oxygen. The carbon monoxide in the smoke is extremely harmful to the human body and can cause poisoning and death in a short period of time. If the concentration of oxygen in the plume is less than 12%, people will suffocate in a short time.
18. Deflagration and explosion. Deflagration is a phenomenon of fire burning that has a great destructive effect on the surrounding facilities. A deflagration wave propagates rapidly and can expand the fire range in a short period. The heat generated by fires can cause gas or dust explosions easily. Incomplete combustion of combustibles leaves flammable and explosive gases and combustible dust, which tend to explode under appropriate conditions.
19. Safe evacuation. Statistics show that safe evacuation directly affects the number of casualties in areas where fires occur. The internal structure of a UEF is complex, with each channel connected to each other and with a few safe exits, making safe evacuation challenging.

Leaf nodes

20. Economic losses. This node is set to three types: Slight (less than RMB 50 million, ~USD 7.2 million), Medium (RMB 50–100 million, ~USD 7.2–14.4 million), Serious (more than RMB 100 million, ~USD 14.4 million) [63].
21. Psychological influence. Fires also cause psychological fear, affect people’s quality of life, and reduce people’s work efficiency. Without timely, effective psychological intervention, psychological trauma becomes increasingly serious, and victims suffer for a long time. However, to the best of our knowledge, no study has incorporated this node into a BN for analysis.
22. Casualties. The status of this node is set to three types: 3 or less dead, or 10 or less seriously injured; 3 or more to 10 dead, or 10 or more to 50 seriously injured; 10 or more to 30 dead, or 50 or more and 100 seriously injured [63].

### 4.2. Establishment of Fuzzy Conditional Probability Table

In order to analyze the causal relationships of UEFs, it is necessary to determine the conditional probability distribution for each node in the Bayesian network. Usually, in CPT construction, if there is a large amount of statistical data, the Closed-Form Learning algorithms [64] and the Max-Min Hill-Climbing algorithms [65] can be used to determine the CPT of each node. However, there is a lack of extensive, detailed accident data for UEF assessment. As an effective and feasible method, expert investigation has been widely used in risk assessment [66]. However, due to the lack of data and changes in the system environment, it is difficult to determine the exact probability. At the same time, experts make subjective judgments on BN nodes, and it is difficult to accurately obtain the influence degree under different states, which leads to a certain ambiguity in the empirical judgment. Therefore, this study considers using fuzzy sets instead of the method of assigning exact probabilities to quantify the CPT judgments of different nodes. Banuls pointed out that three to five experts are enough to make better judgments if there are 10–20 nodes in the network [67]. In this study, we invited 3 experts to perform judgments on the CPTs.

Therefore, CPTs can be determined by fuzzy-set-based expert judgment in the following steps:

1. Division of possibility interval

Dividing the possibility into several intervals can reduce the difficulty of expert judgment and reduce the impact of expert uncertainty. According to the risk possibility classification standard proposed by Zhang et al. [45] and Sun et al. [62], we divided the possibility of causal relationships between node variables into 7 intervals, represented by “1–7”. As shown in Table 1, the ith interval is defined by $\left[a_{i,\;}b_{i}\right]$ together with a mean $m_{i}\left(1\leq i\leq 7\right)$. We set 7 language variables, namely, “very low, low, fairly low, medium, fairly high, high and very high”. This type of correspondence between linguistic variables and probability intervals can facilitate expert questionnaires.

2. Statistical expert opinion

In this study, three experts in the field of safety engineering were invited to score the conditional probability table of each non-root node, and the experts used the possibility partition information proposed in Table 2 for evaluation. Limited by this paper’s length, only the fuzzy conditional probability table of the leaf node casualties is listed, as shown in Table 3.

The leaf node casualties have 4 parent nodes, denoted as $y_{1},\;y_{2},\;y_{3},\;y_{4}$. At the same time, the node has 3 states that are represented by fuzzy numbers 1–3. When scoring by experts, each expert needs to judge the possibility of various states of the child nodes under various combinations of states $y_{1},\;y_{2},\;y_{3},\;y_{4}$. $A_{i}\left(A_{i}=1,2,\cdots ,7\right)$ represents the probability interval of casualties of child nodes in every state. For example, the second row in Table 3, $P\left(S=1|y_{1}=1,\;y_{2}=1,\;y_{3}=1,\;y_{4}=1\right)$, represents the case when the $y_{1},\;y_{2},\;y_{3},\;y_{4}$ state combination is 1, where the node casualties have the possibility of state 1, and the interval is $A_{7}$ = [0.9, 0.95, 1.0].

3. Defuzzification analysis

In this study, a total of 3 experts were invited to participate in the judgment, and 3 FCPTs could be obtained. All the expert scoring data were comprehensively analyzed, and the comprehensive fuzzy number was calculated using Equation (5). A reasonable FCPT can be obtained from the comprehensive evaluation results of experts by using the linear opinion pool method.

(5) $${\tilde{p}}_{1111}^{1}=\frac{\sum_{n=1}^{M}A_{in}}{M}≅\left(a_{1111}^{1},\;b_{1111}^{1},c_{1111}^{1}\;\right)$$

where ${\tilde{p}}_{1111}^{1}$ represents the fuzzy possibility interval, M represents the expert number, and $A_{in}$ represents the nth expert judgment that $p_{1111}^{1}$ is likely to fall into the ith interval.

In order to obtain an accurate CPT of each variable, it is necessary to de-fuzzify the calculation result ${\tilde{p}}_{1111}^{1}$ of Equation (5). In this study, the α-weighted valuation method proposed by Detyniecki and Yager [68] was used to de-fuzzify the fuzzy number and obtain the specific probability value, as shown in Equation (6).

(6) $${\left({\tilde{p}}_{1111}^{1}\right)}^{'}=\frac{a_{1111}^{1}+2b_{1111}^{1}+c_{1111}^{1}}{4}$$

The calculation result of Equation (6) should satisfy the normalization condition so as to obtain the exact normalization result $p_{1111}^{1}$. Equation (7) can be used to normalize the ${\left({\tilde{p}}_{1111}^{1}\right)}^{'}$ results.

(7) $$p_{1111}^{1}=\frac{1}{\sum_{S=1}^{3}{\left({\tilde{p}}_{1111}^{1}\right)}^{'}}\times {\left({\tilde{p}}_{1111}^{1}\right)}^{'}$$

Similarly, the complete CPT of a BN can be calculated according to Equation (6) and Equation (7). Taking the leaf node casualties as an example, the detailed calculation results are shown in Table 4.

## 5. Results and Discussions

**Figure 7:**
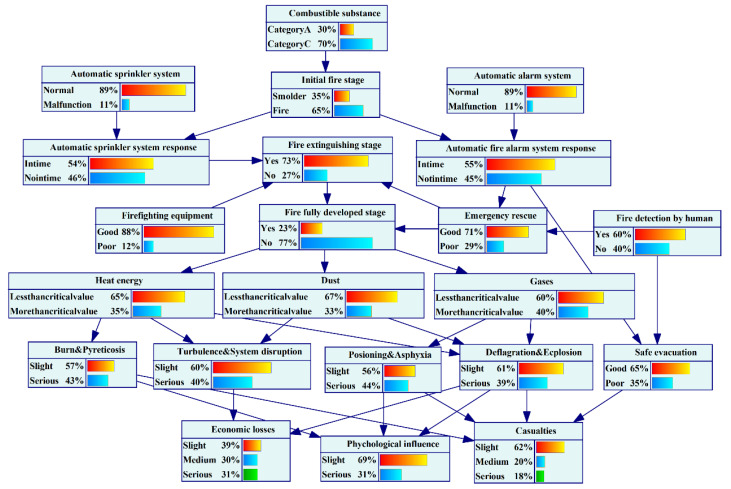
The BN of UEF.

UEFs have many influencing factors that have strong interaction and domino effects, and the resulting disasters have severe consequences. Building such an analysis model enhances insight into UEFs and facilitates the decision-making task. In this paper, we used Bayesian network technology to solve the problem of risk assessment. GeNIE software was applied to learn the structure and parameters of the BN, and the final BN is shown in Figure 7.

### 5.1. The Influence Strength and Sensitivity Analysis

**Figure 8:**
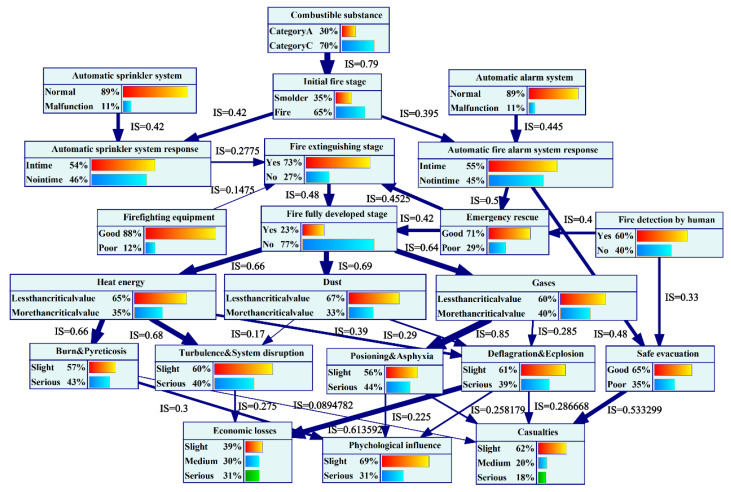
The influence strength of psychological influence.

GeNIE software can use the Euclidean distance formula to calculate the influence intensity of the parent node on the child node and visualize the calculation results [69], as shown in Figure 8.

In Figure 8, the thickness of the directed edge represents the influence intensity, and the thicker the directed edge, the stronger the influence intensity. The value next to the directed edge shows the detailed value of the influence strength. For the node “psychological influence”, the maximum risk transmission path can be determined as follows: automatic fire alarm system→automatic fire alarm system response→emergency rescue→fire extinguishing stage→full fire development stage→heat energy→burn and pyreticosis→psychological influence. Personnel are prone to burns in high-temperature areas and will suffer from fever if they stay for a long time, which will have a great psychological impact on them. Therefore, when a UEF occurs, personnel should take corresponding measures to quickly leave the high-temperature smoke flow area. When a UEF develops to the complete stage, hazards such as heat energy will reach their peak, which is the most dangerous extreme. Therefore, various measures should be taken in the early stage of fire extinguishing to avoid or reduce the development of the UEF to the complete stage. The integrity and normal operation of automatic fire alarm systems play a key role in the early detection of UEFs and the timely evacuation of personnel. The IF value calculated by the BN also reflects this feature. Similarly, for the node “economic losses”, the maximum risk transmission path can be determined as follows: automatic fire alarm system→automatic fire alarm system response→safe evacuation→casualties. By analyzing the risk propagation path, the stability can be greatly improved by cutting off the maximum risk propagation path through various measures (blocking energy transfer, setting security barriers, etc.) in practical projects.

**Figure 9:**
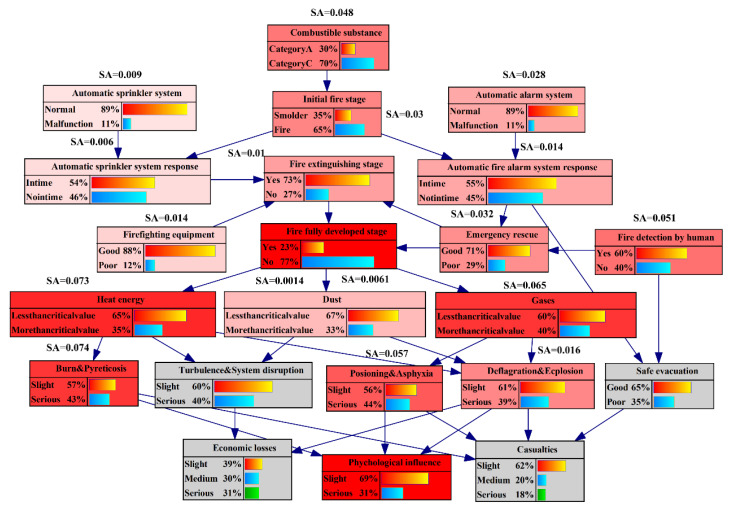
The sensitivity analysis of psychological influence.

In a BN, sensitivity analysis can identify key risk factors, analyze which risk factors are critical in the BN structure, and calculate the degree of impact on target nodes [69], as shown in Figure 9.

In order to analyze the key influencing factors of UEFs more intuitively, this paper set the node “psychological influence” as the target node and analyzed the influence of other variable nodes on this node. In Figure 9, the redder the node color, the higher the sensitivity. The data next to the node show detailed sensitivity values. The results show that the sensitivity of the target node is affected by multiple nodes, and the sensitivity ranking is as follows: burn and pyreticosis > heat energy > gases > full fire development stage > poisoning and asphyxia > personnel fire detection > combustible substance, etc. The most sensitive node is “burn and pyreticosis”. The parent of the “burn and pyreticosis” node is “heat energy”, which is one of the most important contributors to UEFs. In assessing the psychological influence, the assessment should mainly consider the “combustible substance”, “automatic sprinkler system”, “automatic fire alarm system”, and “fir detection by the human” based on “the influence of the transmission of the fully developed stage” on “heat energy”. Therefore, in UEFs, these factors are critical nodes in the BN structure. When the probability of these nodes fluctuates slightly, the probability of the target node will fluctuate sharply. Sensitivity analysis further verified the reliability of the above analysis.

### 5.2. Scenario Analysis

This section, in addition to having underground engineering practitioners and emergency managers construct potential scenarios and predict the probability of each node, mainly discusses three typical accident scenarios to demonstrate the application of the proposed method in a real-world situation. The potential scenarios facilitate the preparation of emergency plans and the design of emergency drills. Comparative analyses of specific scenes before and after the occurrence are shown in the following subsections.

#### 5.2.1. Scenario 1: Categories A and C

Fires caused by the burning of common combustibles, such as wood, paper, sawdust, and coal, are classified as category A fires. The gas products generated by the combustion of category A fires are mainly carbon dioxide and carbon monoxide. The smoke stream contains a small amount of water vapor, methane, and acetylene. Fires that occur in or near electrical equipment are category C fires. Different types of fires produce various levels of damage and have different levels of ease of effective control.

**Figure 10:**
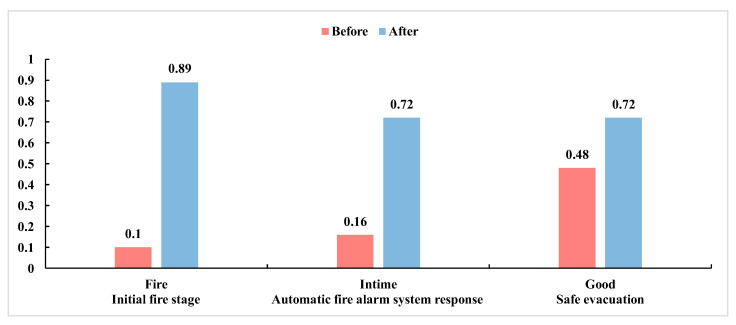
Probabilities before and after Scenario 1 occurrence.

Scenario 1 is activated, and the state of combustibles is changed from category A to category C. The posterior probability of “open fire” increases sharply from 10% to 90%, the posterior probability of “automatic fire alarm system response” increases from 16% to 73%, and the effectiveness of “safe evacuation” increases from 47% to 75%, as shown in Figure 10. The large change in the probability of the “initial fire stage” indicates the importance of these child nodes and needs attention. In general, for category A fires, water and dilute solutions with a high water content can quench or cool combustion materials, thus potentially controlling such fires. The key to controlling category C fires is to cut off the power supply. However, before the power supply is disconnected, personnel must use extinguishing agents for nonconductive fires. Water or dilute solutions with a high water content can be used to extinguish the fire after power disconnection.

#### 5.2.2. Scenario 2: “Heat Energy”, “Burn and Pyreticosis”, “Poisoning and Asphyxia”, “Deflagration and Explosion”, and “Psychological Influence”

**Figure 11:**
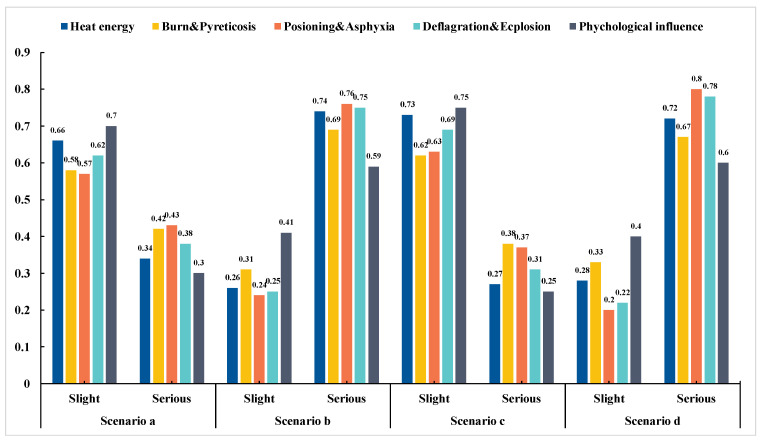
Probabilities before and after Scenario 2 occurrence.

In a fire in an underground space, people in the fire area are all in danger of being affected by smoke. In such cases, smoke, heat energy, and concentrations of toxic and harmful gases are harmful. Based on the proposed structure, this section discusses the following nodes: “burn and pyreticosis”, “poisoning and asphyxia”, “deflagration and explosion”, and “psychological influence”. Several state combinations of these nodes are shown in Table 5, and the estimated probabilities of the results are shown in Figure 11.

In Scenario A, the probability of occurrence of the “slight” state in each variable is higher when wood, paper, garbage, and coal burning are the UEF causes. “Heat energy”, “poisoning and asphyxia”, and “deflagration and explosion” have less harmful consequences. In Scenario B, the posterior probability of the “serious” state of the node “burn and pyreticosis” sharply increases from 0.44 to 0.88 when the fire is not effective; the generated heat energy cannot spread to the surroundings, and the temperature can reach hundreds or even thousands of degrees. People in the high-temperature area are prone to severe burns. When the UEF is caused by electrical equipment, the probability of the “serious” state of each variable rises. For example, in Scenario D, the “serious” posterior probabilities of “poisoning and asphyxia” and “deflagration and explosion” are as high as 0.89 and 0.85, respectively. The concentration of toxic and harmful gases increases, and the concentration of oxygen decreases. Carbon monoxide is extremely harmful to the human body and can poison and kill people in a short time. If the oxygen concentration in the smoke flow is less than 12% (volume concentration), people will suffocate quickly. Methane, acetylene, hydrogen, and similar gases produced by combustion are all flammable and explosive. They are prone to starting serious explosions, resulting in greater fire severities and economic losses. UEFs cause not only human and economic losses but also psychological trauma and other permanent effects on people, but this problem is often overlooked. In Figure 11, when scenarios B and D are activated, the probabilities of generating a “serious” state are as high as 0.79 and 0.82, respectively. After experiencing the sudden, tragic disaster scenarios of UEFs, victims develop, without prompt, effective psychological interventions, psychological trauma, and psychological fear that affect their quality of life and reduce their work efficiency. These psychological effects become increasingly severe and affect patients for a long time. Therefore, governments and relevant departments need to provide a strong guarantee for the treatment of people who have serious psychological fear. Victims should be guided toward treatment and recovery as soon as possible.

#### 5.2.3. Scenario 3: “Combustible Substance”, “Automatic Sprinkler System”, “Automatic Fire Alarm System”, “Firefighting Equipment”, and “Fire Detection by Human”

**Figure 12:**
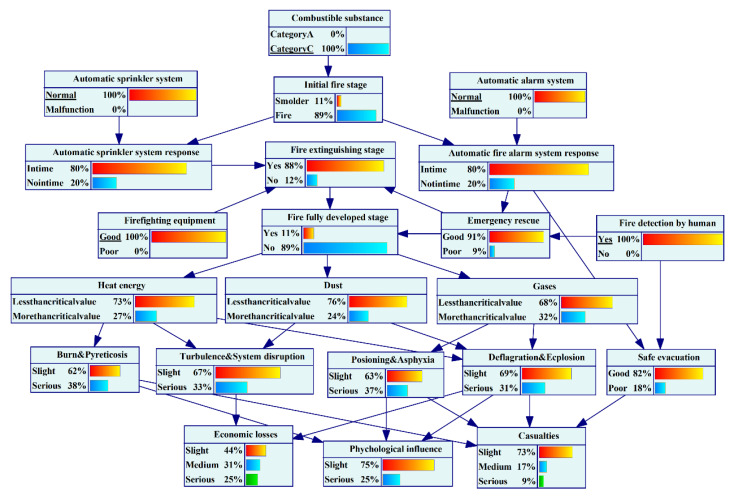
Scenario results with effective fire safety barriers.

**Figure 13:**
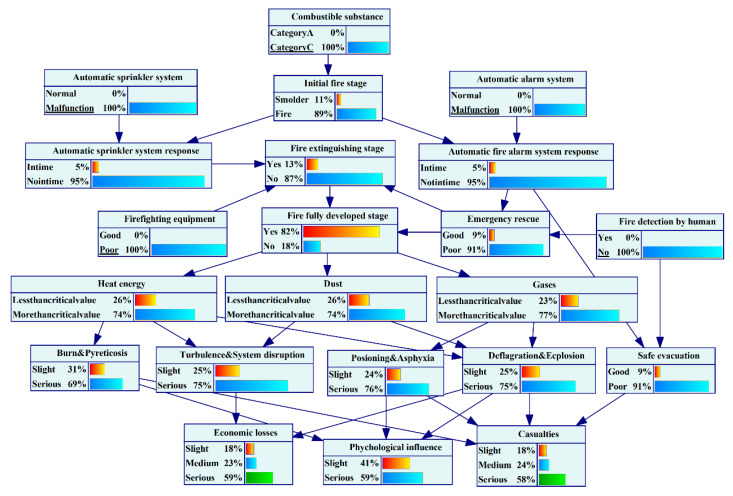
Scenario results with poor fire safety barriers.

Underground engineering spaces are often complicated in structure and layout, with multiple interconnected branch roadways and limited visibility and escape channels. According to the results, the probability that insiders can detect the fire in time is 0.6. When the “combustible substance” is a category A fire and the states of the fire safety barrier nodes are changed (the states of “automatic sprinkler system” and “automatic fire alarm system” change from “normal” to “malfunction”; the “firefighting equipment” status changes from “good” to “poor”; and the status of “fire detection by human” changes from “yes” to “no”), the probability of UEF “casualties” exceeding 10 people rises sharply from 0.05 to 0.7, and the probability of “economic losses” exceeding 100 million is greatly increased from 0.21 to 0.65, as shown in Figure 12 and Figure 13. Therefore, strengthening the construction, management, and maintenance of fire safety barriers and ensuring the normal operation and effectiveness of various safety measures are important for UEF risk prevention and control.

### 5.3. UEF Risk Response Strategies

According to the above analysis, the following countermeasures are put forward for underground engineering fires.

(1) In terms of personnel, people’s emergency self-rescue ability, fire and rescue ability, and crowd-gathering density are issues that need to be focused on. Due to the structural characteristics of underground engineering areas, crowds gather for a long time, the gathering density is high, and emergency evacuation is difficult, so the emergency rescue team should be strengthened to prevent the lack of rescue force when a fire occurs. Professional emergency rescue is an important factor affecting firefighting and safe evacuation. Therefore, there should be enough emergency rescue personnel in the underground engineering area. In addition, the fire risk awareness of underground engineering employees should be strengthened, and fire prevention funds and fire culture publicity should be increased.
(2) Underground engineering is an increasingly complex and functional composite trend; it is necessary to establish fire alarm systems and fire sprinkler systems in the underground engineering area. An intelligent firefighting system with complete function and accurate perception can enable personnel to perceive the fire as soon as possible and then evacuate quickly. At the same time, an alarm system can be connected to the fire alarm system, and the fire professionals can be informed to deal with the fire in time after the alarm.
(3) There are many types of combustibles in underground spaces, which have a certain complexity. Therefore, the storage location and density of articles are particularly important. Fuel storage areas should be marked with noticeable signs and equipped with adequate fire extinguishers. They should be separately stored and well-ventilated.
(4) Management is an important aspect that must be paid attention to. Therefore, it is suggested to strengthen safety inspections in underground engineering areas, actively carry out fire drills, and formulate emergency plans. In addition, the training of fire personnel should be strengthened to raise safety awareness and reduce fire risks.

## 6. Conclusions

Due to the complexity of underground engineering, the high density of personnel, the difficulty of safe evacuation, and the uncertainty of the surrounding environment, the safety risk of underground engineering fires is prominent. Due to the lack of sufficient data, the accurate quantitative evaluation of underground engineering fires is often difficult. In this paper, an evaluation method for underground engineering fires based on EPB-FBN is proposed, which can analyze the cause, occurrence, hazard, product, consequence, and emergency rescue information of UEFs. The main conclusions are as follows.

(1) The EPB model was used to identify the risk factors of UEFs, 22 influencing factors of UEFs were determined, and a reasonable EPB model of UEFs was established. The EPB model was transformed into a BN structure by transforming rules. However, this does not mean that only these 22 variables are associated with UEF risk factors. In this study, in order to improve the efficiency of the analysis, a number of influencing factors were combined. For example, after a UEF, personnel can call a fire alarm number when they spot a fire. Emergency assistance includes the response and arrival time of firefighters. In the future, further research is needed to identify the relatively important risk factors.
(2) By using fuzzy set theory, fuzzy numbers were used to describe the state of variable nodes, a fuzzy conditional probability table was used to describe the logical relationship between UEF nodes, and a fuzzy Bayesian network was established. Experts’ judgment ability and subjectivity were fully considered. By dividing the possibility into intervals, a triangular fuzzy number was selected to represent the linguistic variable of the experts’ judgment. The α-weighted valuation method was used to deblur the fuzzy CPT, and the precise parameters of the fuzzy CPT were obtained. Therefore, the constructed UEF Bayesian network can deal with fuzzy information and uncertain information. This model can be used as a decision tool for UE risk management. Future research should focus on the comparative analysis of different types of fuzzy numbers and different de-fuzzification algorithms.
(3) In addition, through Bayesian sensitivity analysis, influence strength analysis, and deductive reasoning, dynamic evaluation of UEFs can be achieved to identify key risk factors and find the maximum risk chain. This model has high application value in UEF analysis and can be used as a decision-making tool for risk management. With the accumulation of UEF statistics, the next step is to combine data learning with expert judgment and apply more actual data to the model for further study.

## Figures and Tables

**Table 1:** Description and states of Bayesian nodes.

Bayesian Nodes	States of Nodes
Combustible substance	①Category A ②Category C
Automatic sprinkler system	①Normal ②Malfunction
Automatic fire alarm system	①Normal ②Malfunction
Firefighting equipment	①Good ②Poor
Fire detection by human	①Yes ②No
Initial fire stage	①Yes ②No
Automatic sprinkler system response	①In time ②Not in time
Automatic fire alarm system response	①In time ②Not in time
Emergency rescue	①Good ②Poor
Fire extinguishing stage	①Slight ③Serious
Full fire development stage	①Yes ②No
Heat energy	①Less than critical value ②More than critical value
Dust	①Less than critical value ②More than critical value
Gases	①Less than critical value ②More than critical value
Burn and pyreticosis	①Slight ②Serious
Turbulence and system disruption	①Slight ②Serious
Poisoning and asphyxia	①Slight ②Serious
Deflagration and explosion	①Slight ②Serious
Safe evacuation	①Good ②Poor
Economic losses	①Slight ②Medium ③Serious
Psychological influence	①Slight ②Serious
Casualties	①Slight ②Medium ③Serious

**Table 2:** Division of possibility intervals.

$\mathrm{Possibility}\;\mathrm{Interval}\;(A_{i})$	Linguistic Variable	$\mathrm{Lower}\;\mathrm{Bound}\;(a_{i})$	$\mathrm{Mean}\;(m_{i})$	$\mathrm{Upper}\;\mathrm{Bound}\;(b_{i})$
1	Very low	0	0.05	0.1
2	Low	0.1	0.2	0.3
3	Fairy low	0.3	0.35	0.4
4	Medium	0.4	0.5	0.6
5	Fairly high	0.6	0.65	0.7
6	High	0.7	0.8	0.9
7	Very high	0.9	0.95	1.0

**Table 3:** FCPT of leaf node casualties in the DAG.

$y_{1}$	$y_{2}$	$y_{3}$	$y_{4}$	$P\left(S=1|y_{1},\;y_{2},\;y_{3},\;y_{4}\right)$	$P\left(S=2|y_{1},\;y_{2},\;y_{3},\;y_{4}\right)$	$P\left(S=3|y_{1},\;y_{2},\;y_{3},\;y_{4}\right)$
				$A_{i}$	$A_{i}$	$A_{i}$
1	1	1	1	$A_{7}$	$A_{1}$	$A_{1}$
1	1	1	2	$A_{6}$	$A_{1}$	$A_{1}$
1	1	2	1	$A_{7}$	$A_{2}$	$A_{1}$
1	1	2	2	$A_{6}$	$A_{2}$	$A_{1}$
1	2	1	1	$A_{6}$	$A_{2}$	$A_{1}$
…	…	…	…	…	…	…
2	1	2	2	$A_{5}$	$A_{2}$	$A_{1}$
2	2	1	1	$A_{6}$	$A_{3}$	$A_{1}$
2	2	1	2	$A_{1}$	$A_{4}$	$A_{4}$
2	2	2	1	$A_{1}$	$A_{5}$	$A_{3}$
2	2	2	2	$A_{1}$	$A_{1}$	$A_{7}$

**Table 4:** Investigation and analysis results for node casualties.

Node	Expert A			Expert B			Expert C			Fuzzification			Defuzzification	Normalization
P1111 S = 1	0.9	0.95	1	0.9	0.95	1	0.9	0.95	1	0.9	0.95	1	0.95	0.90
P1111 S = 2	0	0.05	0.1	0	0.05	0.1	0	0.05	0.1	0	0.05	0.1	0.05	0.05
P1111 S = 3	0	0.05	0.1	0	0.05	0.1	0	0.05	0.1	0	0.05	0.1	0.05	0.05
P1112 S = 1	0.7	0.8	0.9	0.7	0.8	0.9	0.9	0.95	1	0.766666667	0.85	0.933333333	0.85	0.85
P1112 S = 2	0	0.05	0.1	0	0.05	0.1	0.1	0.2	0.3	0.033333333	0.1	0.166666667	0.1	0.10
P1112 S = 3	0	0.05	0.1	0	0.05	0.1	0	0.05	0.1	0	0.05	0.1	0.05	0.05
…	…	…	…	…	…	…	…	…	…	…	…	…	…	…
P2221 S = 1	0	0.05	0.1	0	0.05	0.1	0	0.05	0.1	0	0.05	0.1	0.05	0.05
P2221 S = 2	0.6	0.65	0.7	0.4	0.5	0.6	0.4	0.5	0.6	0.466666667	0.55	0.633333333	0.55	0.55
P2221 S = 3	0.3	0.35	0.4	0.3	0.35	0.4	0.4	0.5	0.6	0.333333333	0.4	0.466666667	0.4	0.40
P2222 S = 1	0	0.05	0.1	0	0.05	0.1	0	0.05	0.1	0	0.05	0.1	0.05	0.04
P2222 S = 2	0	0.05	0.1	0	0.05	0.1	0.1	0.2	0.3	0.033333333	0.1	0.166666667	0.1	0.10
P2222 S = 3	0.9	0.95	1	0.9	0.95	1	0.7	0.8	0.9	0.833333333	0.9	0.966666667	0.9	0.86

**Table 5:** Initial setup of Bayesian nodes.

Bayesian Nodes	Setup of Bayesian Nodes			
	Scenario A	Scenario B	Scenario C	Scenario D
Combustible substance	Category A	Category A	Category C	Category C
Automatic sprinkler system	Normal	Malfunction	Normal	Malfunction
Automatic fire alarm system	Normal	Malfunction	Normal	Malfunction
Firefighting equipment	Good	Poor	Good	Poor
Fire detection by human	Yes	No	Yes	No
